# Blooming Phenograms, Pollen Production, and Pollen Quality during Storage of Pistachio Cultivars in New Mediterranean Growing Areas

**DOI:** 10.3390/plants13182606

**Published:** 2024-09-18

**Authors:** Giuseppe Lillo, Claudio Calia, Danilo Cice, Milena Petriccione, Salvatore Camposeo

**Affiliations:** 1 Department of Soil, Plant and Food Science, University of Bari Aldo Moro, Via Amendola, 165/A, 70126 Bari, Italy; g.lillo8@studenti.uniba.it (G.L.); salvatore.camposeo@uniba.it (S.C.); 2 Council for Agricultural Research and Economics, Research Centre for Olive, Fruit and Citrus Crops, Via Torrino, 3, 81100 Caserta, Italy; danilo93mhr@gmail.com (D.C.); milena.petriccione@crea.gov.it (M.P.)

**Keywords:** *Pistacia vera*, ‘Peter’, ‘Randy’, ‘Golden Hills’, ‘Lost Hills’, ‘Napoletana’, germinability, viability

## Abstract

Pistachio (*Pistacia vera* L.) is a dioecious, anemophilous, and drought-resistant fruit tree species. It is cultivated in new Mediterranean areas, including the regions of southern Italy (Apulia and Basilicata). It has been estimated that over 40,000 ha are suitable for pistachio cultivation in areas infected by *Xylella fastidiosa* subsp. *pauca*. As a newly introduced species, knowledge of its biological reproductive behaviors in its new areas of spreading is essential for appropriate agronomic planning and management. This two-year study (2022 and 2023), carried out in the countryside of Stigliano (MT, Italy), had the objective of evaluating the flowering phenograms, pollen production, and assessing protocols for the conservation and extension of pollen viability, of the most widespread cultivars. A slight delay was observed in the blooming phenograms, compared to other cultivation Mediterranean areas, such as Spain or Sicily. Furthermore, the overlap between female and male phenograms was partial. No significant differences were observed in the polliniferous aptitude of the two male cultivars. Among the different protocols tested, the pollen storage at 33% relative humidity and a temperature of −80 °C maintained the pollen germinability above 50% for up to three weeks. These findings highlight the importance of controlled environmental conditions in preserving pollen viability over extended periods, providing valuable insights for agricultural and botanical research that relies on maintaining pollen viability for breeding and genetic studies.

## 1. Introduction

The pistachio is a dioecious, anemophilous, and drought-resistant tree species, known for its adaptability to arid climates [1]. It is cultivated in the Mediterranean basin (Spain, Italy, Turkey, Iran) and western America (California and Arizona). Over the past five years, the main producers of pistachios have been the USA, Iran, China, and Turkey, with Iran and Turkey leading in terms of the largest areas under cultivation [2]. In the last five years in Italy, there has been a notable and steady increase in both areas and total production, probably linked to the increase in the typicality of this product in certain areas and the consequent increase in demand [3]. Nowadays, in Italy, the most cultivated cultivar is ‘Napoletana’ (syn. ‘Bianca’), as it is listed in the production specifications of the ‘Bronte green pistachio’ and ‘Raffadali pistachio’ Protected Designation of Origin (PDO); however, relatively few cultivars are currently used in worldwide pistachio production such as ‘Kerman’, ‘Lost Hills’, ‘Golden Hills’, ‘Aegina’, ‘Larnaka’, ‘Siirt’, ‘Red Aleppo’, and ‘Mateur’ [4,5]. Cultivars are generally grafted onto different rootstocks belonging to different species of the genus *Pistacia* (*P. atlantica*, *P. terebinthus*, *P. integerrima*) or, currently, onto patented rootstocks obtained from interspecific hybrids such as UCB1 (*P. atlantica* × *P. integerrima*), PG1 (*P. integerrima* Stewart), and PG2 (*P. atlantica × P. integerrima*) [6,7].

In the last decade, due to the nutritional properties of nuts, there has been an increase in demand, with a consequent price increase (EUR 12/kg Italian price). Pistachio cultivation is spreading to new Mediterranean growing areas, including the peninsular regions of southern Italy (Apulia and Basilicata). Moreover, the discovery of *Xylella fastidiosa* subsp. *pauca* in the Apulian region has posed a significant threat to olive groves and the wider agro-environmental system [8,9,10]. Although pistachio trees, like almonds, are theoretically susceptible to this bacterium, no infected or symptomatic pistachio trees have been reported in these affected areas. This suggests that pistachio may be a viable alternative fruit crop in regions traditionally dominated by olive cultivation, such as Apulia, which are also part of the agricultural biodiversity enhancement plan [10]. In a recent study, it was estimated that more than 40,000 hectares are suitable for pistachio cultivation in areas infected by *Xylella* [10]. To optimize pistachio cultivation in new areas, it is essential to understand the specific agronomic requirements and biological behavior of pistachio tree [11,12].

Pistachio, being a dioecious species, relies heavily on effective pollination strategies for successful production. As a wind-pollinated species, the selection of appropriate female cultivars and pollinizers within the orchard is crucial for maximizing yields. In commercial orchards, different combinations of female and male cultivars are selected to ensure synchronized flowering, and male trees are placed among the female ones in a ratio of 4% to 15% [13]. One of the critical factors in this selection process is the synchronization of blooming dates between male and female trees, which ensures efficient natural pollination. Flowering and subsequent pollination are critical stages in pistachio cultivation, as they have a direct impact on crop yield [14]. Typically, commercial pistachio orchards contain around 12% male trees, with one male tree generally planted for every eight female trees. In regions without dominant winds, a higher proportion of male trees can be beneficial, particularly when planted along plot boundaries to improve pollen dispersal [15]. Pistachio inflorescences are panicles of several hundred flowers, with each male flower containing five or six anthers [16]. As pistachios are not attractive to insect pollinators, they rely entirely on wind for pollination. Although artificial pollination is not widely practiced, it can be a viable option where natural pollination is insufficient or compromised [1]. Most male pistachios trees tend to bloom earlier than female trees, often releasing pollen before the female flowers are receptive [17]. Artificial pollination can, therefore, be a valuable practice in orchards to improve fruit set when natural pollination is insufficient or unreliable, as is currently the case for other fruit tree species such as kiwi, date palm, walnut, hazelnut, and olive [18,19,20,21,22,23]. A further significant challenge in pistachio cultivation is represented by the limited storability of pollen. Despite several protocols studied, up to now, the viability and germinability of pistachio pollen cannot be extend beyond two months [16,24]. For an accurate assessment of pollination potential, it is crucial to measure both pollen viability and germinability, as these parameters directly impact the effectiveness of both natural and artificial pollination strategies.

This research aimed to investigate three main aspects related to pistachio flowering and pollen management in a new Mediterranean growing area: (I) to establish the detailed blooming phenograms, (II) to evaluate the polliniferous aptitude of the male cultivars ‘Randy’ and ‘Peters’, and (III) to assess storage protocols for the conservation and extension of pollen viability. The results would be very useful for optimizing pollination strategies and improving the overall productivity and sustainability of pistachio cultivation in new environments.

## 2. Materials and Methods

### 2.1. Orchard and Experimetal Design

**Figure 1:**
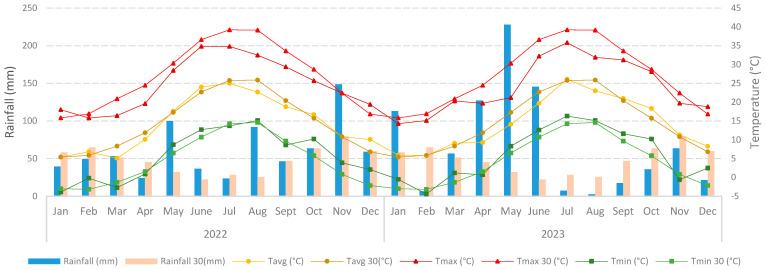
Weather conditions (average, maximum, and minimum temperatures and rainfall) over the last 30 years compared with the 2022 and 2023 crop years.

This two-year study was carried out in Stigliano (Matera, Basilicata region, southern Italy; 40°20′22.32″ N; 16°14′1.66″ E; 278 m a.s.l.). on a loamy-clay soil with a low amount of skeleton. This area is characterized by a typical semiarid Mediterranean climate associated with mild, moist winters and dry and hot summers, with a higher rainfall concentration during fall and spring. Figure 1 shows the climatic data for the two growing seasons, compared with the 30-year climatic averages. The climatic data of the two crop years were obtained from the Italian Civil Protection, while the 30-year averages were obtained from NASA data [25]. The average annual rainfall in the year 2022 was 736 mm, while in 2023 it was 825 mm (Figure 1).

The experiment was conducted during the 2022 and 2023 growing seasons, in an orchard of 8.5 ha, planted in 2017 consisting of 6–7-year-old trees planted with a spacing of 6 m × 6 m apart (278 trees per hectare). The orchard included three female cultivars: ‘Golden Hills’, ‘Lost Hills’, and ‘Napoletana’; and two male cultivars: ‘Peter’ and ‘Randy’; all cultivars grafted onto UCB1 rootstock. The male trees constitute 11% of the total, with one male tree for every nine female trees. Chilling units (CUs) were calculated using the UTAH method, based on climatic data from a weather station in Stigliano operated by the civil protection authority [26,27]. In 2022, the area accumulated 2073 CUs while in 2023, it received 1500 CUs. These levels of CUs are sufficient for the successful cultivation of the cultivars ‘Randy’ and ‘Peter’ [26,28,29]. The experimental design was the complete randomization block, with three replications.

### 2.2. Blooming Phenogram and Pollen Production

**Figure 2:**
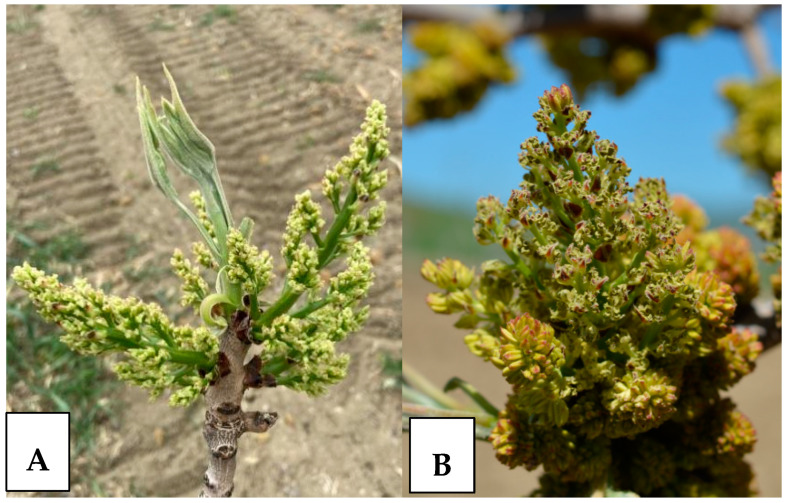
Female inflorescences (‘Napoletana’ (**A**)) and male inflorescences (‘Randy’ (**B**)) in anthesis.

Blooming phenograms were assessed on three randomly selected trees per plot, for each male and female cultivar. For each tree, eight branches were labeled, positioned at four sides of the canopy. For each side of the canopy, two canopy sections (lower and upper level) were identified. In order to accurately determine the phenological stage of blooming, data collection occurred every 48–72 h. Each flower was scored with either a 0 or +1 point (Figure 2): male inflorescences were assigned a +1 when they were fully extended and began to release pollen, while female inflorescences received a +1 when they were fully extended and the stigma was receptive [30,31,32,33]. For each cultivar, blooming phenogram started and ended when 10% and 90% of flowers were open, respectively; full bloom was achieved when 50% of flowers were open.

In order to estimate the number of flowers per inflorescence and assess polliniferous aptitude, five additional male trees were randomly selected. For each male cultivar, ten inflorescences were collected from each tree (50 inf); so five flowers from each inflorescence were randomly selected (250 flowers), grouped in sets of ten flowers, and placed in Falcon tubes (25 Falcons). The flowers (*n* = 10) were then kept at room temperature for 48 h to allow for complete pollen release through natural dehiscence [28,34,35,36,37,38,39,40].

Once the flowers were fully dehiscent, 4 mL of 5% Tween20^®^ aqueous solution was added to each Falcon tube. The solution was then subjected to ultrasonic vibration to facilitate the release of pollen from the anthers and to ensure thorough dispersion in the solution. Finally, two drops of this solution were then transferred to each chamber of a Bürker hemocytometer, and the pollen grains were counted under the microscope for each chamber (18 chambers). The following formula was used to determine the number of pollen grains per flower, considering the number of pollen grains observed in the chambers and the dilution factor [38]:

$$A=\frac{n\;*\;B}{N}$$

where

*A* = number pollen grains per flower;

*n* = number of pollen grains counted in each cell;

*B* = fraction of suspension retained by each chamber about the original amount of suspension in each vial. In this case, the dilution factor was 0.1 mL, dilution 1:40,000;

*N* = number of flowers in each vial (10 flowers/Falcon).

**Figure 3:**
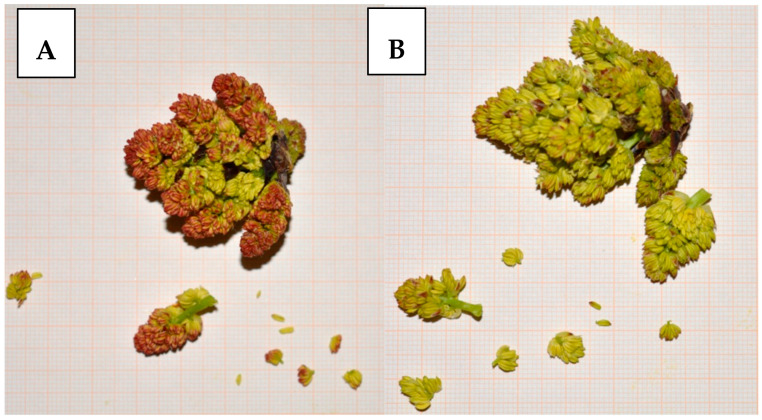
Counting flowers per inflorescence in ‘Peter’ (**A**) and in ’Randy’ (**B**).

To scale up to the number of pollen grains per inflorescence, we firstly quantified the number of flowers on each inflorescence on 50 inflorescence per cultivar (Figure 3). Therefore, we multiplied the total number of flowers per inflorescence by the average number of pollen grains per flower calculated by the formula.

### 2.3. Pollen Storage

Different protocols were applied for the pollen conservation [16,41,42]. Pollen viability and germinability were monitored. Pollen samples were collected from randomly selected inflorescences for each cultivar and subsequently placed on glossy paper at room temperature for about 24 h to dry. Three storage protocols were applied on pollen at approximately 30% humidity:

- PROTOCOL A—refrigerated at +5 °C (PA);
- PROTOCOL B—frozen at −20 °C (PB);
- PROTOCOL C—cryopreserved at −80 °C (PC).

**Figure 4:**
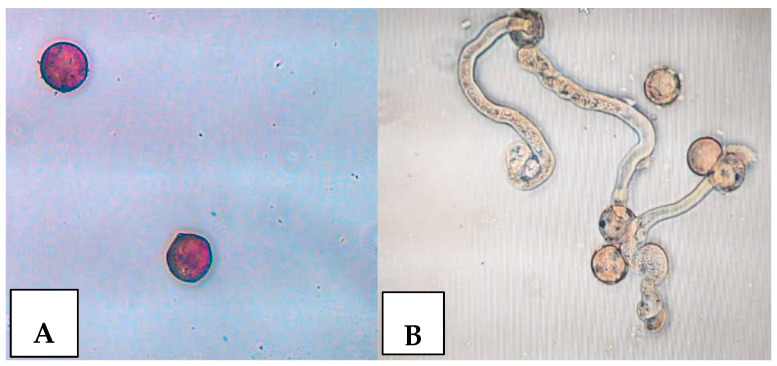
(**A**) Pollen viability test (stained with 1% TCC); (**B**) pollen germinability.

Viability tests were performed on each protocol with TTC staining (1% *v*/*v* 2,3,5-Triphenyltetrazolium chloride, 30% *w*/*v* sucrose) [16,43]. Germinability was assessed using the hanging drop method (20% sucrose 40 ppm boric acid, incubated at 20–25 °C for 24 h) [43,44]. A pollen grain was considered germinated when the pollen tube exceeded the diameter of the pollen grain itself [42] (Figure 4).

The tests were conducted eight times: fresh pollen (0 h) and after 48 h, 72 h, 1 week, 2 weeks, 3 weeks, 4 weeks, and 2 months. For each timing and storage protocol, pollen quality tests were assessed in triplicate.

### 2.4. Experimental Design and Statistical Analysis

For both pollen production and storage, a two-way ANOVA was applied. For pollen storage, the independent variables were ‘exposure time’ and ‘temperature’, while for pollen production, the variables were ‘year’ and ‘cultivar’. The post hoc tests were LSD and Bonferroni (*p* < 0.05). Rstudio 4.1.3 software was used.

## 3. Results

### 3.1. Blooming Phenograms

**Figure 5:**
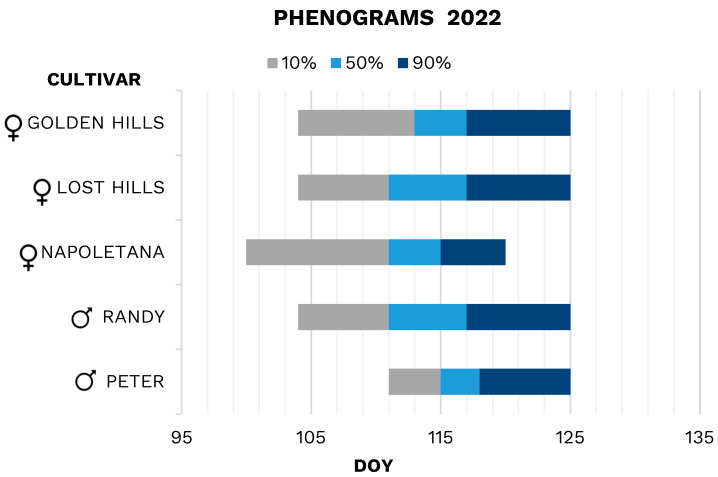
Blooming phenograms of the female and male pistachio cultivars in 2022.

In 2022 (Figure 5), the flowering of female cultivars ‘Lost Hills’ and ‘Golden Hills’ started on DOY 104; ‘Golden Hills’ lasted about 9 days (from 104 to 113), and ‘Lost Hills’ about 7 days (from 104 to 111). The full bloom for ‘Golden Hills’ lasted 4 days (from 113 to 117), ‘Lost Hills’ 6 days (from 111 to 117); for the two Californian cultivars, the bloom ended on 5 May (DOY 125). Female cultivar ‘Napoletana’ started to flowering on 10 April (DOY 100), the early flowering lasted 11 days, until 21 April (DOY 111), the full bloom 4 days (from 111 to 115); the flowering ended on 30 April (DOY 120). ‘Napoletana’ started flowering about 4 days earlier than the other two female cultivars, but the full bloom coincided (Figure 5). For the male cultivar ‘Randy’, flowering started on 14 April (DOY 104), the early flowering lasted 7 days, while the full bloom lasted 6 days (from 111 to 117) and ended on 5 May (DOY 125). ‘Peter’ started blooming later than ‘Randy’, on 21 April (DOY 111); the first phase of flowering lasted 4 days, while the full bloom lasted only 3 (from 115 to 118), the bloom ended on 5 May (DOY 125; Figure 5). ‘Randy’ has a very extensive and early full bloom compared to ‘Peter’.

‘Randy’ blooming overlapped completely (100%) with the female cultivars ‘Lost Hills’ and ‘Golden Hills’ and 80% with ‘Napoletana’. ‘Peter’ overlapped 72% with the female cultivars ‘Lost Hills’ and ‘Golden Hills’ and only 45% with ‘Napoletana’.

**Figure 6:**
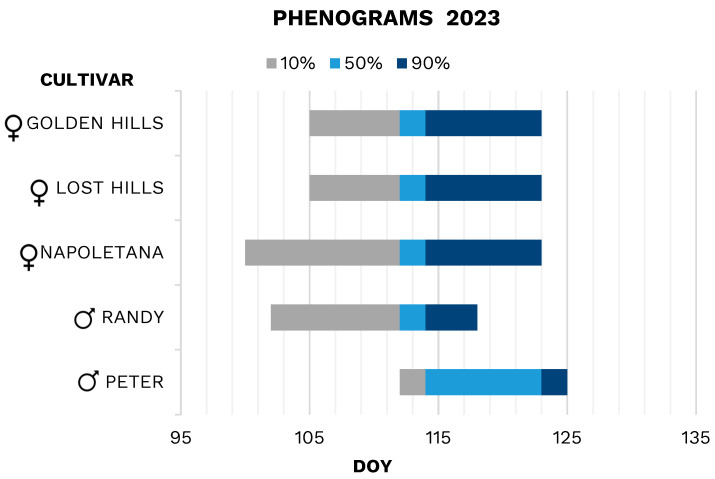
Blooming phenograms of the female and male pistachio cultivars in 2023.

In 2023 (Figure 6), ‘Lost Hills’ and ‘Golden Hills’ had simultaneous flowering; for both cultivars, the onset flowering was 15 April 2023 (DOY 105), the early flowering lasted about 7 days, while the full bloom 2 days (from 112 to 114), and the flowering ended on 3 May (DOY 123). For the cultivar ‘Napoletana’, the flowering started on 10 April (DOY 100), early flowering lasted 12 days (until 22 April), full bloom 2 days (from 112 to 114), and flowering ended on 3 May (DOY 123). For the early male ‘Randy’, flowering started on 12 April (DOY 102), the early flowering lasted 10 days, while the full bloom lasted 2 days (from 112 to 114), ending on 28 April (DOY 118). The later male ‘Peter’ started flowering on 22 April (DOY 112), the early flowering lasted until 24 April (DOY 114; 2 days), while the full bloom lasted about 9 days (from 114 to 123), and ended on 5 May (DOY 125; Figure 6).

Blooming phenogram of cultivar ‘Randy’ was 70% overlapped with ‘Golden Hills’ and ‘Lost Hills’ ones, and 70% with ‘Napoletana’. ‘Peter’ blooming phenogram overlapped with the phenograms of the cultivar ‘Lost Hills’ and ‘Golden Hills’ by 61%, while the overlap with ‘Napoletana’ was only 47% (Figure 6).

### 3.2. Pollen Production

Mean pollen production per flower was 1.31 × 10^5^ and 1.52 × 10^5^ and the mean number of pollen grains per inflorescence was 4.54 × 10^7^ and 5.10 × 10^7^ in 2022 and 2023, respectively, without statistical difference between the two growing years (Table 1). The mean number of flowers per inflorescence was 340, irrespective of cultivar and year. On the contrary, significant differences were observed between cultivars and their interaction with the growing years. Indeed, ‘Randy’ produced more pollen with respect to ‘Peter’, with 1.58 × 10^5^ pollen grains per flower and 5.26 × 10^7^ per inflorescence and 1.25 × 10^5^ pollen grains per flower and 4.38 × 10^7^ per inflorescence, respectively (Table 1).

### 3.3. Pollen Viability and Germinability during Storage

The data on pollen quality were collected in only 2022, because in 2023 the male inflorescence was affected by mold contamination, resulting in very low pollen values that were excluded from the analysis. The pollen of ‘Randy’, stored with PA (5 °C), showed a reduction in viability after 72 h, with viability rates decreasing from initial levels to between 86% and 73.2% (Table 2). In contrast, pollen stored under PB (−20 °C) maintained better quality for a longer period, with viability dropping to 71.8% after 2 weeks, comparable to the viability observed after 72 h under protocol A. After one week, pollen viability was 66.8%, 76%, and 73.3% for PA, PB, and PC, respectively. After 2 weeks, viability was 58.8% for PA (−27.2% from the initial level), 71.8% for PB (−14.2% from the initial level), and 68.1% for PC (−17.9% from the initial level). After 3 weeks, pollen viability was relatively higher under PC (63.6%), which was 9.3% higher than in PB and 11% higher than in PA. However, viability significantly decreased after 4 weeks, reaching 34.6%, 37.1%, and 51.4%, for PA, PB, and PC, respectively. Finally, after 2 months of storage, viability of the pollen were reduced to zero.

The initial pollen germinability of ‘Randy’ (Table 2) was about 76%. After 48 h, it decreased to 69.9%, 74.6%, and 73.8% for PA, PB, and PC, respectively. A significant reduction was observed after one week of storage, with a decrease of 12.4% in PA, 8% in PB, and 7% in PC. After two weeks, germinability fell below the 50% threshold for PA (46%), while it remained above this threshold for PB (64.4%) and PC (62.9%). After three weeks, only the germinability in PC (53.4%) remained just above the 50% threshold, whereas PA and PB had decreased to 26.7% and 41%, respectively. At four weeks, germinability ranged from a maximum of 25% to a minimum of 11.4%. After 2 months, the pollen was no longer germinable.

The initial pollen germinability of ‘Randy’ (Table 2) was about 76%. After 48 h, it decreased to 69.9%, 74.6%, and 73.8% for PA, PB, and PC, respectively. A significant reduction was observed after one week of storage, with a decrease of 12.4% in PA, 8% in PB, and 7% in PC. After two weeks, germinability fell below the 50% threshold for PA (46%), while it remained above this threshold for PB (64.4%) and PC (62.9%). After three weeks, only the germinability in PC (53.4%) remained just above the 50% threshold, whereas PA and PB had decreased to 26.7% and 41%, respectively. At four weeks, germinability ranged from a maximum of 25% to a minimum of 11.4%. After 2 months, the pollen was no longer viable. For ‘Peter’, pollen stored in PA showed a viability reduction from the initial 100% to 82.3% after 48 h. In contrast, pollen stored in PB (−20 °C) and PC (−80 °C) had higher viability, at 93.2% and 96.1%, respectively (Table 3). After 72 h, viability decreased to 64.3% for PA, 81.7% for PB, and 92% for PC. After one week, a reduction of 42.6% for PA, 34% for PB, and 27.9% for PC was observed, compared to the initial values. The viability decreased after 2 weeks to 46.2% for PA (−53.8% from the initial level), whereas PB presented an unusual value of 73.2%, and PC presented a value of 66.2% (−33.8% from the initial level). After 3 weeks, viability remained just above the threshold only in PC (54.6%), whereas PB showed a value of 44.1% and PA of 23.6%. The viability dropped significantly after 4 weeks, to 43.3%, 29.4%, and 19.2% for PC, PB, and PA, respectively. However, the complete loss of pollen viability was reached after two months (Table 3).

The pollen germinability of ‘Peter’ (Table 3) presented initial values of about 91%. After 48 h, it decreased to 74.4%, 87.4%, and 89.7% for PA, PB, and PC, respectively. A significant reduction was already observed after 72 h of storage, with values dropping to 54% for PA, 73.4% for PB, and 85.7% for PC. After one week, germinability fell below the 50% threshold for PA (42%), while it remained above this threshold for PB (54.9%) and PC (63.4%). After two weeks, only the germinability in PC (54.4%) remained just above the 50% threshold, whereas PA and PB had values of 35.2% and 41.9%, respectively. At four weeks, germinability ranged between a maximum of 39.9% (PC) and a minimum of 17.9% (PA). After two months, the pollen was no longer viable (Table 3).

**Figure 7:**
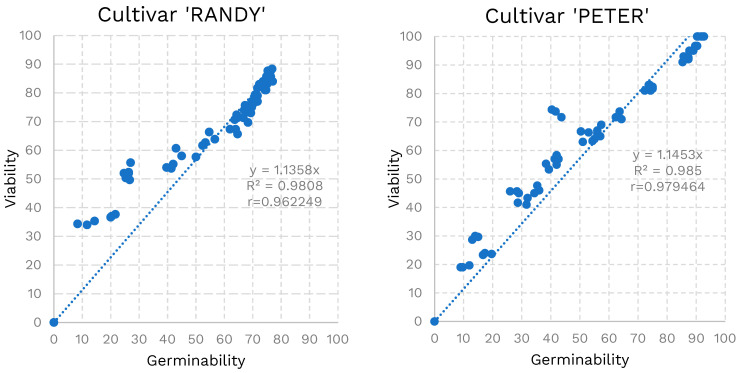
Linear regressions between pollen germinability and viability for cultivars ‘Randy’ and ‘Peter’.

The regressions between the two pollen quality indices (Figure 7) showed a good correlation (r = 0.96) for the cultivar ‘Randy’. However, at lower values (under 40%) of germinability, viability appeared to overestimate the actual germinability. For cultivar ‘Peter’, the regression analysis highlighted a higher correlation (r = 0.98), and viability appeared to overestimate the actual germinability less.

## 4. Discussion

The overlapping of blooming phenograms is one of the most important aspects of the design and selection of cultivars in orchard production [1,14]. In the growing areas examined, flowering phenograms occurred slightly later compared to other Mediterranean regions, such as Spain or Sicily, and even more so compared to Californian regions [30,45]. Male cultivars ‘Randy’ and ‘Peter’ exhibited a delayed flowering period of 7 to 10 days. The female cultivars ‘Lost Hills’ and ‘Golden Hills’ showed a delayed flowering period of 4 to 10 days. The cultivar ‘Napoletana’ in environments like Spain has an earlier flowering period of a few days compared to the area considered in this study. The impact of the climatic factors on blooming phenograms is confirmed by Lodolini et al. [46], suggesting that the thermal sums required for flowering are achieved later than in the Mediterranean or Californian environments. With climate change, male cultivars, with mild winters, might experience greater delays in flowering, leading to reduced synchronization between female cultivars and pollinizers. In the long run, this could lead to reduced production compared to its potential, with significant risks of compromising investment. ‘Randy’ has an earlier flowering than ’Peter’ [28,34] likely due to its lower need for cooling units. These traits may allow ‘Randy’ to better adapt to the rising average temperature than to ‘Peter’ in this area. ’Napoletana’ was the earliest to flower (DOY 100), followed by the other two female cultivars, ‘Lost Hills’ and ‘Golden Hills’, which flower simultaneously (DOY 104). The flowering duration for these varieties is about 20 days, and full flowering can vary considerably. In fact, in the year 2022, full flowering for ‘Lost Hills’ lasted 6 days, while the full flowering of ‘Napoletana’ and ‘Golden Hills’ was 4 days. In 2023, the duration of full flowering was identical for these cultivars. The cultivar ‘Randy’ was found to be perfectly synchronized with the flowering phenograms of both cultivars ‘Lost Hills’ and ‘Golden Hills’. Additionally, ‘Randy’ was well aligned with both the start and full flowering period of ‘Napoletana’. In contrast, the phenogram of ‘Peter’ (DOY 111–112) coincided with the full flowering of the three female cultivars, but its full flowering period aligned with their end of flowering. ‘Randy’ emerges as the most effective male cultivar for the female cultivars examined. It was perfectly synchronized with the flowering phenograms of the cultivars ‘Golden Hills’ and ‘Lost Hills’ across both years and it partially (70%) overlapped with cultivar ‘Napoletana’. Introducing a smaller proportion of ‘Peter’ could be effective for covering the later stages of flowering, although its overlap with the female flowering phenograms is reduced from 30% to 50% for the Californian cultivars and approximately 40% for the Italian cultivar.

Pistachio bloom dates were strongly correlated with chill accumulation, with low chill periods delaying bloom dates for several pistachio cultivars, as observed by Elloumi et al. [47]. Furthermore, no cultivars flower if pistachio cultivars do not reach a minimum chill threshold need to bloom [48].

The Bürker chamber allowed us to estimate the amount of pollen produced by each cultivar. Both cultivars ‘Randy’ and ‘Peter’ showed no statistically significant differences in pollen production per inflorescence. Given the critical role of selecting and arranging pollinizer cultivars for optimizing pollination, evaluating polliniferous aptitude is essential. This assessment helps estimate the pollen output of a pollinizer and identify the most suitable male cultivar for specific conditions [38,45].

Aldahadha et al. [49] demonstrated significant variability in vitro pollen germination percentage for six pistachio cultivars among them. Fresh pollen from the cultivar ‘Lazaourdi’ exhibits the highest germination rate at 69.7%, followed by ‘Aschouri’ and ‘Boundiki’, then ‘Batouri’ and ‘Marawhi’. The cultivar ‘Nab-El Jamal’ shows the lowest rate at 40.3%. Acar et al. [50] reported that the male pistachio cultivars ‘Atli’, ‘Uygur’, and ‘Kaska’ generally had better pollen germination than their F1 hybrids under in vitro conditions. Contrary to Polito and Luza [42], who noted a loss of pistachio pollen germinability after several days, Vaknin and Eisikowitch [51] found that fresh pollen lost most of its germinability within hours, with the highest germination rate at 76.7%. Günver-Dalkılıç and Dayı Doğru [52] observed that pistachio pollen germination ratios ranged from 78.22% to 63.29% on the first day at room temperature, dropping to 55.83% to 43.26% by the second day when stored in a refrigerator. After one month of refrigerated storage, all cultivars showed zero germination, in agreement with Ateyyeh [16]. Although ‘Peter’ maintained a higher germinability and viability of fresh pollen and in the first 48 h of storage, ‘Randy’ was more suitable for storage, as it maintained higher viability and germinability values for a longer period than ‘Peter’. Under the PA protocol (5 °C), ‘Randy’ exhibited a rapid decline in viability, dropping from an initial value of 86% to 73.2% after 72 h. In contrast, the PB protocol (−20 °C), provided better preservation of pollen quality, with viability decreasing to 71.8% after 2 weeks (similar values were reached after 72 h in the PA). However, in both tested cultivars, the PC protocol (33% HR and −80 °C) allowed us to preserve pollen quality over time. The results obtained from the different preservation protocols were obtained in both cultivars and overlap with the results reported in the literature [38]. Despite several attempts to extend the pollen viability, it was not possible to exceed four weeks of storage. This limitation is attributed to the unique traits of pistachio pollen, unlike other tree species that maintain the viability of their pollen for a longer time under the same storage conditions. Pistachio pollen lacks special grooves and pollen-kitt molecules that help retain cellular hydration, making it particularly susceptible to dehydration [53,54]. Consequently, the vegetative and germinative cells of the pollen granule undergo deplasmolysis, leading to pollen death [54]. This presents challenges for the storage and subsequent use of pistachio pollen. The same pollen retention difficulties were observed for other anemophilous pollinated nut species such as walnut and chestnut [55,56], while other entomophilous-pollinated fruit species such as the almond, date palm, and kiwi have shown long-lasting pollen retention [16,57,58]. In vitro pollen germination is highlighted as a valuable technique for understanding the physiological and biochemical requirements for successful pollen germination and pollen tube growth. It is also a reliable method for assessing the viability of both fresh and stored pollen. However, the pollen of pistachio is noted to be challenging to germinate in vitro [49]. Additionally, the correlation index between the two pollen quality indicators was high (98.5% in ‘Peter’ and 96.2% in ‘Randy’; Figure 7). This suggest that viability, as measured by TTC staining, closely approximates actual germinability. Therefore, TTC staining provides a reliable estimate of pollen viability with about 96% accuracy, offering a quicker alternative to germinability tests.

## 5. Conclusions

These are the first results regarding the floral biology of pistachio cultivars grown in a new Mediterranean environment. The male cultivar ‘Randy’ seemed to be the most effective pollinizer, demonstrating perfect synchronization (100%) with female cultivars ‘Lost Hills’ and ‘Golden Hills’ and achieving a substantial overlap (70–80%) with cultivar ‘Napoletana’. Additionally, while ‘Peter’ can be useful for extending the flowering period of female cultivars in certain years, ‘Randy’ has the most polliniferous attitude in terms of pollen production. In contrast, the viability and germinability of pollen of ‘Peter’ are highest in the first 48 h after storage. Moreover, the storage at −80 °C is the most effective method, preserving over 50% germinability and viability for up to three weeks. This research represents a first step in the industrial application of artificial pollination for pistachios, providing crucial insights that can transform production practices. These findings not only support the expansion of pistachio growing into new Mediterranean areas but also set the stage for future innovations in orchard management and crop optimization.

## Figures and Tables

**Table 1:** Quantity of pollen grains per flower, number of flowers per inflorescence, and quantity of pollen grains per inflorescence. Interaction between the two variables (Y × Cv) are also shown. For each parameter, the means followed by the same letter are not significantly different at *p* = 0.05 (LSD test). Standard errors are also reported. Statistical difference at * *p* ≤ 0.05, ** *p* ≤ 0.01.

	Pollen Grains/Flower		Flowers/Inflorescence		Pollen Grains/Inflorescence	
2022	1.31 × 10^5^ ± 2.51 × 10^4^	a	345.83 ± 14.16	a	4.54 × 10^7^ ± 08.64 × 10^6^	a
2023	1.52 × 10^5^ ± 5.03 × 10^4^	a	339.78 ± 16.01	a	5.10 × 10^7^ ± 15.20 × 10^6^	a
	ns		ns		ns	
‘Peter’	1.25 × 10^5^ ± 2.45 × 10^4^	b	350.83 ± 09.68	a	4.38 × 10^7^ ± 08.08 × 10^6^	b
‘Randy’	1.58 × 10^5^ ± 4.64 × 10^4^	a	334.78 ± 15.18	a	5.26 × 10^7^ ± 14.57 × 10^6^	a
	*		ns		*	
Y × Cv	**		ns		**	

**Table 2:** Viability and germinability of cultivar ‘Randy’ pollen during storage under different storage protocols (PA: 33% RH 5 °C; PB: 33% RH −20 °C; PC: 33% RH −80 °C). Means followed by the same letter in the rows do not differ significantly at *p* = 0.05 (LSD test). Standard errors are also reported.

Cultivar ‘RANDY’												
Timing	Viability						Germinability					
	PA		PB		PC		PA		PB		PC	
0 h	86.0 ± 2.2	a	85.8 ± 1.8	a	85.8 ± 1.8	a	75.1 ± 1.6	a	76.1 ± 0.4	a	76.0 ± 0.9	a
48 h	76.1 ± 4.9	cd	82.2 ± 1.0	b	81.7 ± 1.2	b	69.9 ± 1.5	cd	74.6 ± 0.5	a	73.8 ± 1.3	ab
72 h	73.2 ± 1.6	de	76.0 ± 1.0	cd	78.4 ± 1.3	c	67.3 ± 0.9	de	69.8 ± 0.2	cd	71.4 ± 0.5	bc
1 week	66.8 ± 1.0	f	74.7 ± 1.0	de	73.3 ± 0.6	de	63.6 ± 1.4	f	68.1 ± 0.8	d	69.0 ± 0.6	cd
2 weeks	58.8 ± 1.6	h	71.8 ± 0.5	e	68.1 ± 3.7	f	46.0 ± 3.6	h	64.4 ± 0.2	ef	62.9 ± 5.9	f
3 weeks	52.6 ± 3.0	i	54.3 ± 0.8	i	63.6 ± 2.5	g	26.7 ± 0.3	j	41.0 ± 1.2	i	53.4 ± 1.2	g
4 weeks	34.6 ± 0.7	j	37.1 ± 0.5	j	51.4 ± 1.0	i	11.4 ± 3.0	l	20.7 ± 0.9	k	25.4 ± 0.8	j
2 months	0.0 ± 0.0	k	0.0 ± 0.0	k	0.0 ± 0.0	k	0.0 ± 0.0	m	0.0 ± 0.0	m	0.0 ± 0.0	m

**Table 3:** Viability and germinability of cultivar ‘Peter’ pollen during storage under different storage protocols (PA: 33% RH 5 °C; PB: 33% RH −20 °C; PC: 33% RH −80 °C). Means followed by the same letter in the rows do not differ significantly at *p* = 0.05 (LSD test). Standard errors are also reported.

Cultivar ‘PETER’												
Timing	Viability						Germinability					
	PA		PB		PC		PA		PB		PC	
0 h	100.0 ± 0.0	a	100.0 ± 0.0	a	100 ± 0.0	a	91.2 ± 1.3	a	91.1 ± 1.1	a	91.4 ± 0.7	a
48 h	82.3 ± 0.7	d	93.2 ± 1.6	c	96.1 ± 1.0	b	74.7 ± 0.6	d	87.4 ± 0.2	bc	89.7 ± 0.7	ab
72 h	64.3 ± 0.1	f	81.7 ± 1.2	d	92 ± 1.00	c	54.0 ± 2.6	f	73.4 ± 1.0	d	85.7 ± 0.3	c
1 week	57.4 ± 0.8	g	66.2 ± 2.8	f	72.1 ± 1.4	e	42.0 ± 0.7	g	54.9 ± 2.2	f	63.4 ± 1.0	e
2 weeks	46.2 ± 1.3	i	73.2 ±1.4	e	66.2 ± 1.1	f	35.2 ± 0.8	h	41.9 ± 1.7	g	54.4 ± 3.6	f
3 weeks	23.7 ± 0.3	l	44.1 ± 2.1	j	54.6 ± 1.1	h	17.9 ± 1.6	k	27.9 ± 1.6	j	39.9 ± 1.9	g
4 weeks	19.2 ± 0.4	m	29.4 ± 0.7	k	43.3 ± 2.3	j	10.2 ± 1.6	m	14.0 ± 1.0	l	30.7 ± 2.0	i
2 months	0.0 ± 0.0	n	0.0 ± 0.0	n	0.0 ± 0.0	n	0.0 ± 0.0	n	0.0 ± 0.0	n	0.0 ± 0.0	n

## Data Availability

The data presented in this study are available in the article.
